# Applications of indocyanine green (ICG) fluorescence technology in open surgery: preliminary experience in pediatric surgery

**DOI:** 10.3389/fsurg.2023.1238487

**Published:** 2023-08-16

**Authors:** Ciro Esposito, Benedetta Lepore, Mariapina Cerulo, Fulvia Del Conte, Vincenzo Coppola, Giovanni Esposito, Roberto Carulli, Francesca Carraturo, Maria Escolino

**Affiliations:** ^1^ Pediatric Surgery Unit, Department of Translational Medical Science, University of Naples “Federico II”, Naples, Italy; ^2^ CEINGE Advanced Biotechnologies Center Franco Salvatore scarl, Naples, Italy

**Keywords:** rubina lens, fluorescence, open surgery, nir, ICG, children

## Abstract

**Background:**

Indocyanine green fluorescence technology (ICG) in pediatric minimally invasive surgery has undergone an important improvement in the last 5 years. However, its use in open surgery is still limited. In this paper, we aim to report our preliminary experience with Rubina® lens ICG fluorescence technology in combination with the IMAGE1 S™ system from KARL STORZ in open excision of masses in children.

**Methods:**

The records of 18 patients undergoing open surgery for head, neck and thorax masses between September and November 2022 were retrospectively reviewed. Rubina® lens ICG fluorescence technology system was used in all the cases. In 10 cases we adopted the holding arm system and in 8 cases the hand-held technique. Data about patients' demographics, surgery and outcomes were collected and analyzed through the following criteria: mass localization, intraoperative time (min), ICG administration (ml), intraoperative complications, postoperative complications.

**Results:**

A total of 18 patients were operated: 4 thyroglossal duct cysts, 3 supraorbital cysts, 2 neck masses, 2 pre-auricular and 2 scalp cysts, 2 gynecomastias, 2 lymphangiomas, 1 nose mass. In all the cases, intralesional injection of 0.5–1 ml of ICG solution was performed peri-operatively. Mean operative time was 58.4 min (35–134 min). Postoperative complications included seroma formation in 2 cases. Surgical pathology reports confirmed complete mass excision in all the cases.

**Conclusion:**

Based on our preliminary experience, ICG fluorescence guided surgery using Rubina® lens system was very helpful also in open surgery procedures. Rubina® lens system permits to have a very low complication rate, a time-saving surgery, a real time reliability of anatomic structures and an excellent clinical safety. In our experience, holding arm system seems more comfortable than hand-held system. However, further cases need to be performed to evaluate the exact role and to identify new indications of this technique in open pediatric surgical procedures.

## Introduction

Most head, neck and thorax masses occurring in children are of either inflammatory/infectious, congenital, or of neoplastic origin (1). The first ones can be the result of reactive lymphadenopathy or infectious lymphadenitis. Common congenital developmental masses include thyroglossal duct cysts, branchial cleft cysts, dermoid cysts, vascular malformations, hemangiomas, lymphangiomas or gynecomastia. Other lesions are represented by common benign neoplastic lesions and include pilomatrixomas, lipomas, fibromas, neurofibromas, and salivary gland tumors (2). Excision of head, neck and thorax masses is recommended to confirm the diagnosis and to prevent complications (e.g.,: exact histology, potential growth, secondary infection) (3). For most lesions, the treatment of choice remains complete open excision (4, 5). However, sometimes it is difficult to exactly identify the lesions margins, above all because the masses have the same color of surrounding tissues. Singer et al. demonstrated the presence of procedure-related complications for hospital readmission after pediatric neck mass surgery, including postoperative neck mass, wound issues, and infection (6). In case of boys with gynecomastia, surgical gland resection is indicated for patients with long-lasting disease when mass does not regress spontaneously or following medical therapy, or for psychological reasons (7–9). The use of Near-infrared fluorescence (NIRF) imaging with indocyanine green (ICG) has been adopted in pediatric minimally invasive surgery (MIS) in order to improve intra-operative visualization of anatomic structures, facilitate surgery and reduce postoperative complications (10). However, few experiences of ICG use in open surgery has been reported until now in particular in pediatric age. For this reason, we decided to adopt Rubina® lens ICG fluorescence technology system for open surgical mass excision of pediatric patients and evaluate its outcomes.

## Materials and methods

**Figure 1:**
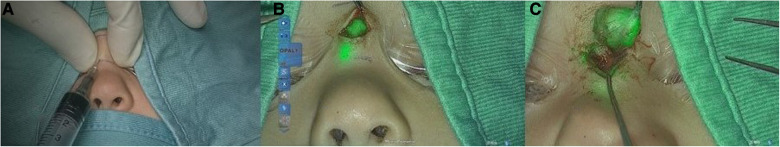
Injection of ICG in the nasal cyst (**A**); visualization of the cyst with ICG (**B**); cyst removal (**C**).

**Figure 2:**
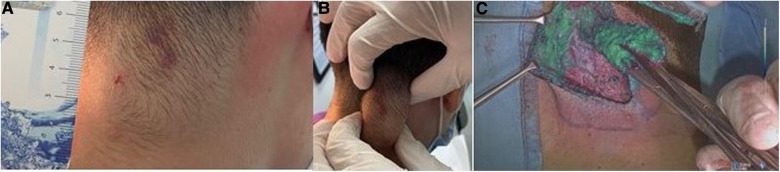
Measuring the lymphangioma (**A**); lymphangioma margins demarcation (**B**); visualization of the lymphangioma with ICG (**C**).

**Figure 3:**
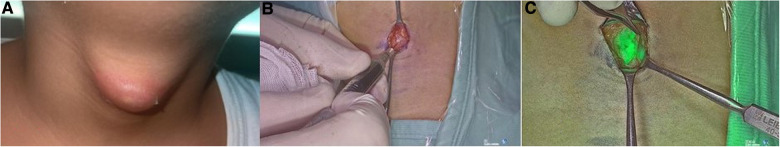
Visualization of thyroglossal duct cyst (**A**); injection of ICG in the cyst (**B**); visualization of the cyst with ICG (**C**).

**Figure 4:**
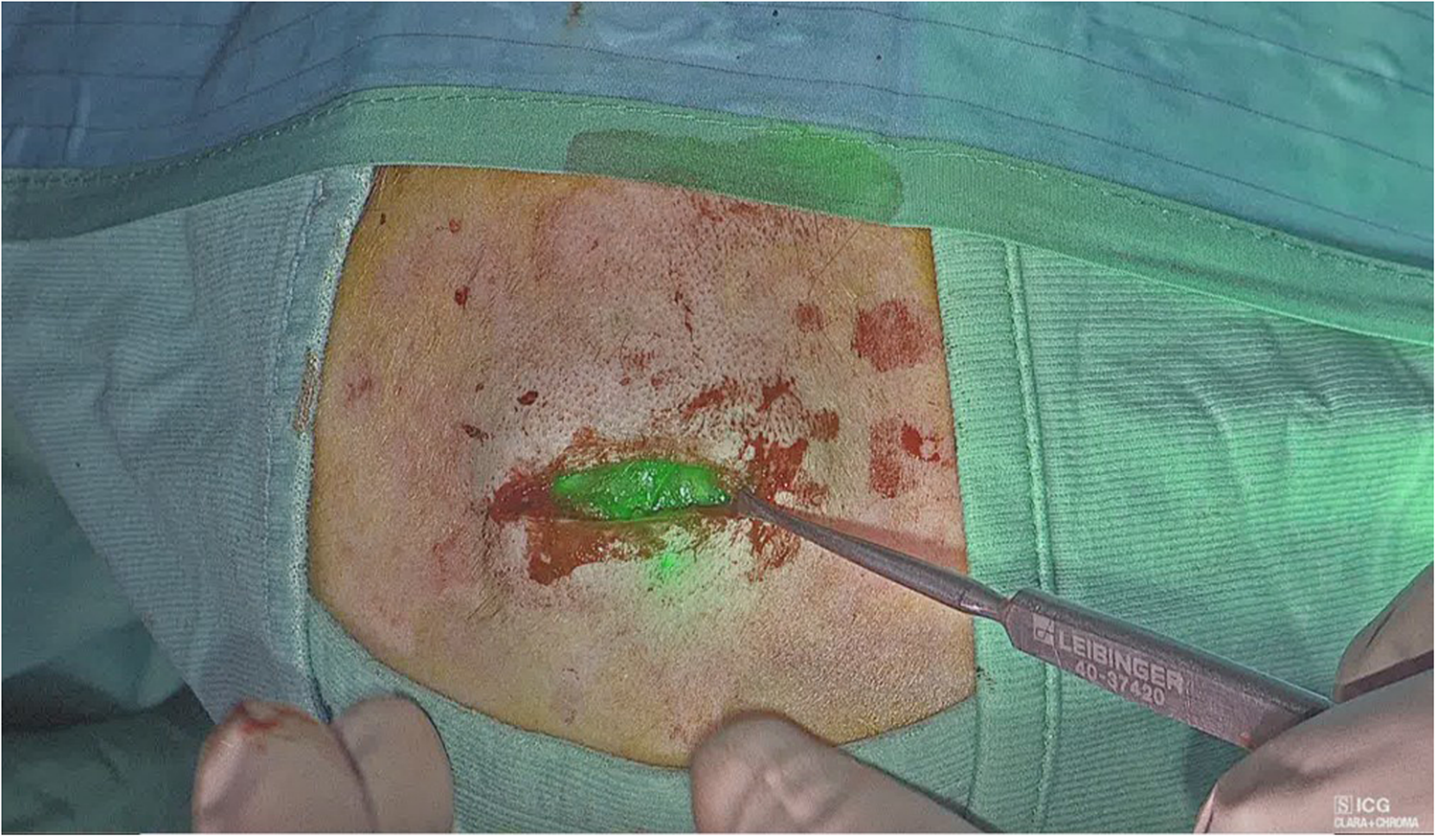
Visualization of ICG in the skull cyst.

**Figure 5:**
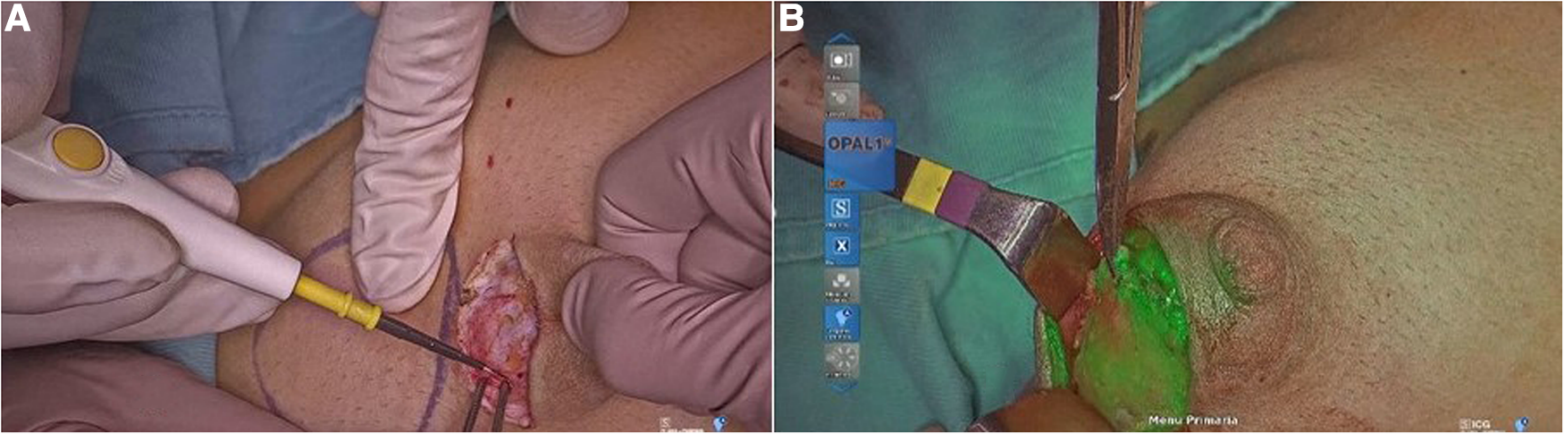
Mammary gland excision in gynecomastia (**A**); visualization of ICG in the mammary gland (**B**).

We retrospectively analyzed the data of pediatric patients undergoing surgical mass excision in our center with open Rubina® lens ICG fluorescence technology in combination with the IMAGE1 S™ system from KARL STORZ from September 2022 to November 2022. During this period, a total of 18 pediatric patients with head neck and thoracic masses were treated surgically. There were 11 boys and 7 girls, with a median age of 6 years (1–15). Patient characteristics are showed in Table 1. All the patients had a minimum follow-up of 2 months and attended the outpatient department at 1 week and 1 month post-operatively. All the patients received general anesthesia. The surgery involved a complete open excision of head, neck and thoracic masses with minimal margins and a primary closure of the defect using separated stitches (Figures 1–4). For gynecomastia, subcutaneous mastectomy through a peri-areolar access has been used (Figure 5). Intralesional injection of 0.5–1 ml of ICG solution (in dependence of mass size) during the operation using a 25-gauge needle was performed. 2 ml of solution were injected for thyroglossal duct cysts. 5 ml of solution were injected for neck masses, which presented a diameter of 5 cm–6 cm. For gynecomastia cases, ICG solution was injected intradermally into the periareolar region of the breast. The solution was obtained using a vial of ICG (5 mg/ml) diluted with 5 ml of sterile water, for a total of 10 ml of solution. In every case, the NIRF signal lasted for the whole time of the surgery.

**Table 1:** Patients demographics.

Parameter	Patients (*n* = 18)
M/F, *n*/*n*	11/7
Median patient age, years (range)	6 (1–15)
Follow up, months (range)	2 (2–4)

**Figure 6:**
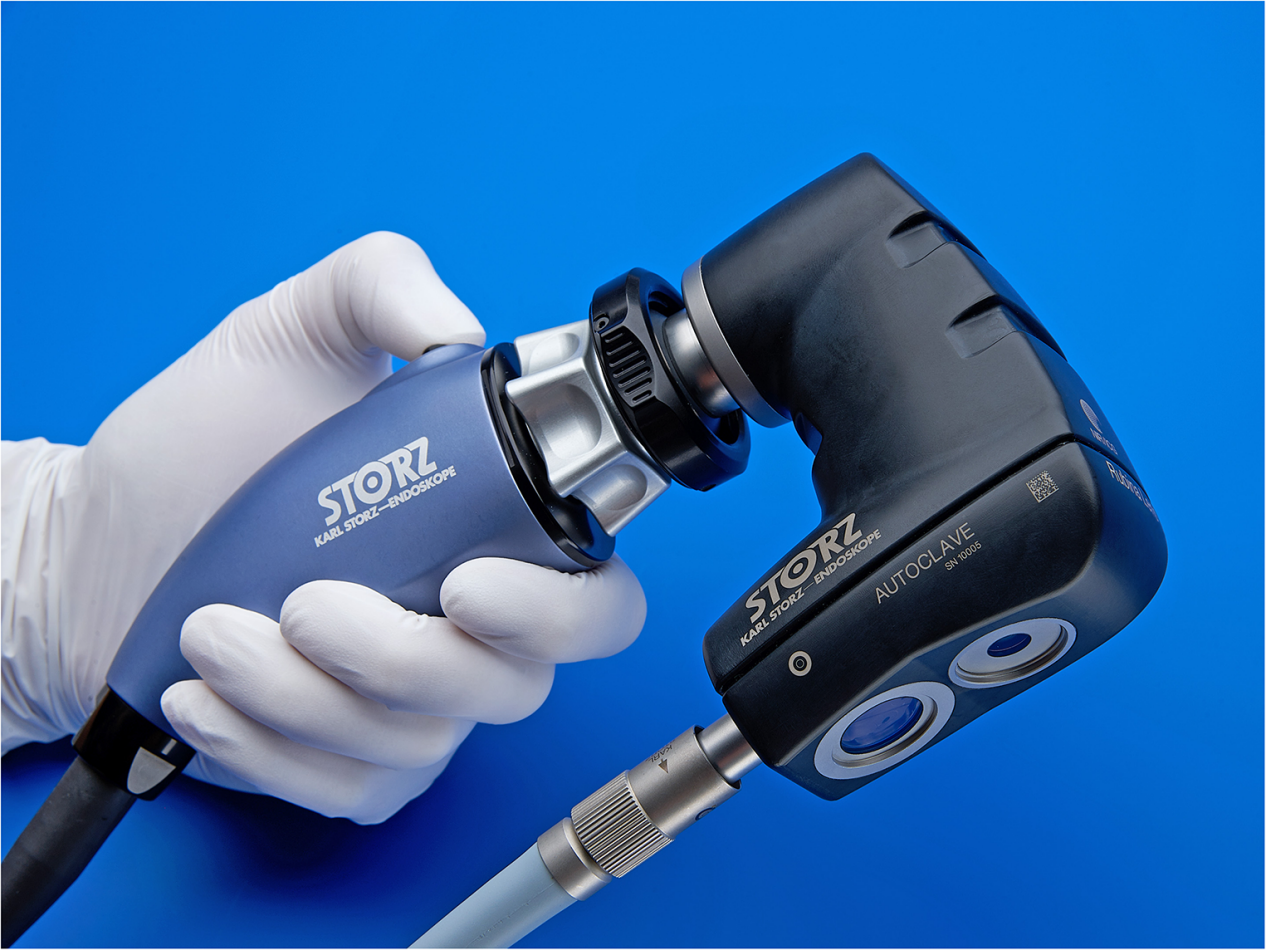
RUBINA® lens system held by hand.

**Figure 7:**
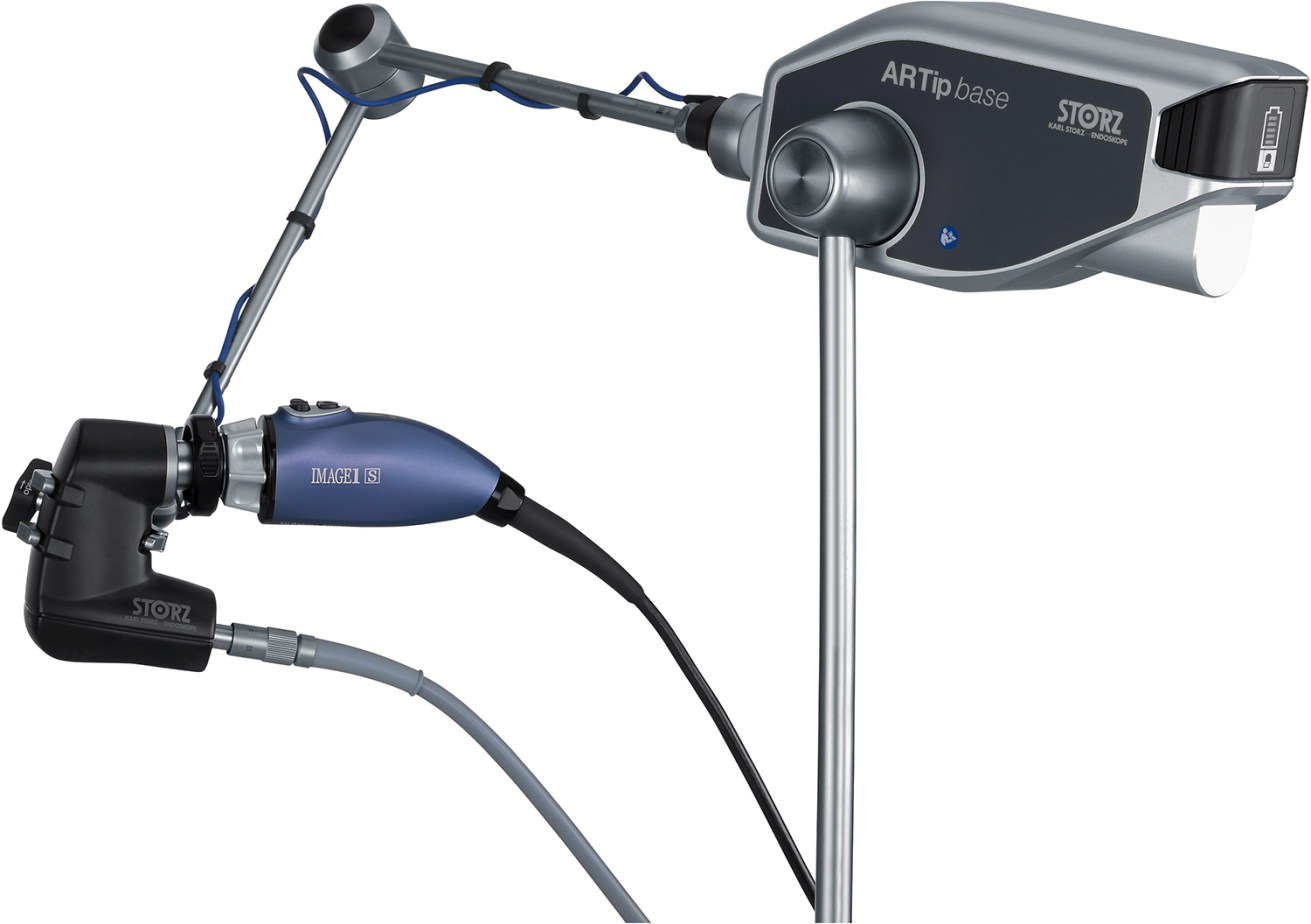
RUBINA® lens system held by the holding arm.

**Figure 8:**
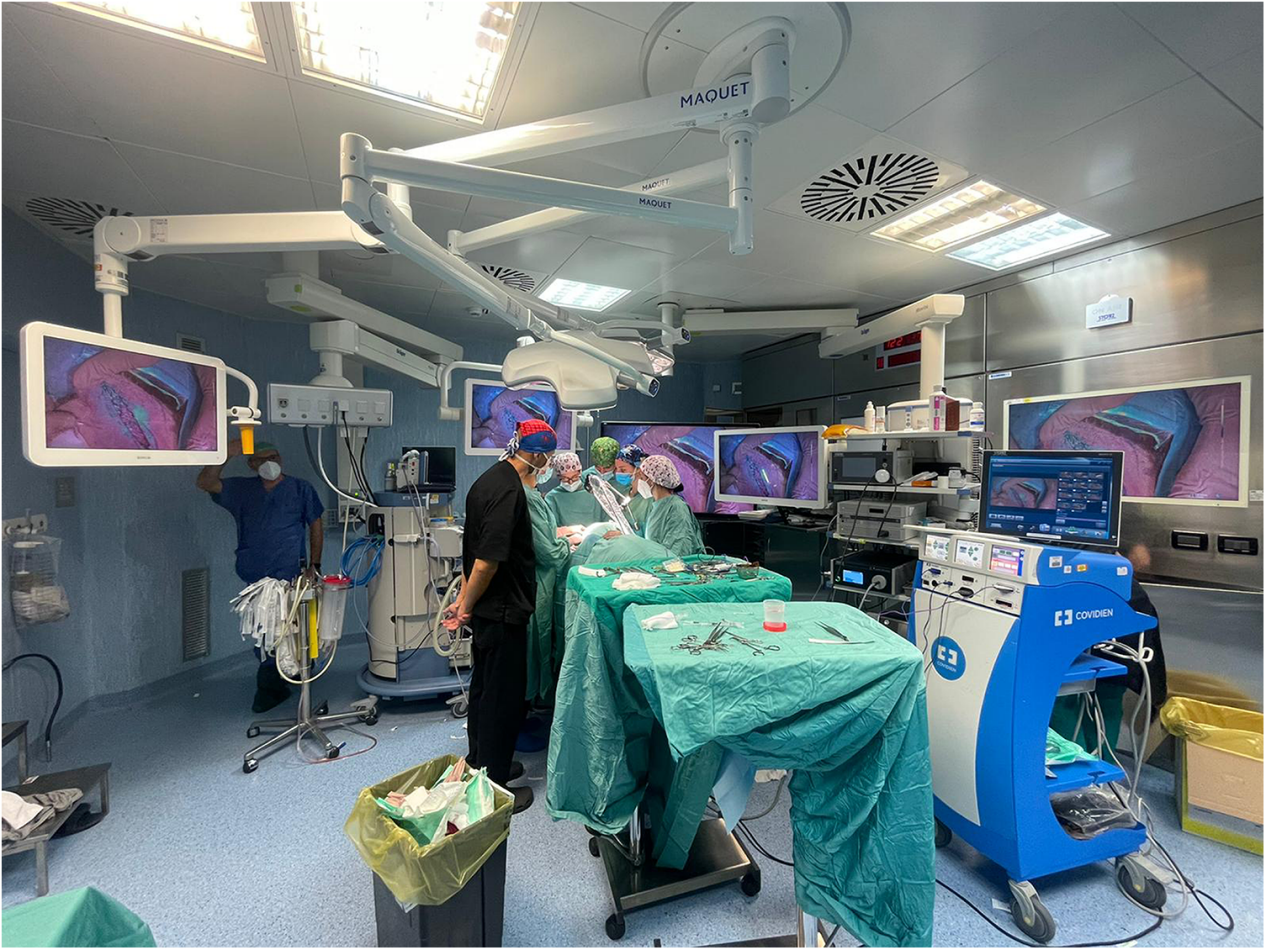
An example of multimedial operating room set for the rubina® lens system.

In our study, we used Rubina® lens system in 2 ways: hand-held technique (Figure 6), when the assistant holds the camera in the hands during surgery, and holding arm technique (Figure 7), when a metallic arm attached to the table is used to hold the camera. The working distance of camera when the holding arm system is in place is up to 13 in. away from the patient, with field-of-view adjustable from 4.7 in. (12 cm) to 2 in. (5 cm) simply by moving the device toward or away from the surgical field (Figure 8). Data about patients' demographics, surgery and outcomes were collected and analyzed through the following criteria: mass localization, intraoperative time (min), ICG administration (ml), intraoperative complications, postoperative complications.

The study received the appropriate Institute Review Board (IRB) approval. Informed consent to participate in the study was obtained from all participants or their parents or legal guardian in the case of children under 16.

## Results

Table 2 shows patients demographics. A total of 18 patients were operated: 4 thyroglossal duct cysts, 3 supraorbital cysts, 2 neck masses, 2 pre-auricular cysts, 2 scalp cyst, 2 gynecomastias, 2 lymphangiomas, 1 supranasal cyst. In all the cases, intralesional injection of 0.5–1 ml of ICG solution during the operation using a 25-gauge needle was performed. No adverse reaction to ICG was reported. Table 3 shows surgical details of mass excision with Rubina® Lens. Mean intra-operative time was 58.4 min (35–134 min). The visualization with Rubina® lens always showed clear differentiation. No intraoperative complications were reported. Postoperative complications were present only in two cases (neck mass and thyroglossal duct cyst) and manifested 3 weeks after surgery: they involved the formation of a seroma, which was managed through fluid aspiration in the outpatient setting. Complete mass excision was confirmed by the surgical pathology reports. Margin enlargement was never required. At follow up of 4 months, no patients presented recurrence of the mass or other complication related to the procedure.

**Table 2:** Patients characteristics.

*ID*	Type of mass	Gender	Age
1	Supranasal cyst	F	5
2	Neck mass	F	7
3	Neck mass	F	8
4	Scalp cyst	M	7
5	Scalp cyst	M	9
6	Supraorbital cyst	F	2
7	Supraorbital cyst	M	2
8	Supraorbital cyst	M	6
9	Thyroglossal duct cyst	F	3
10	Thyroglossal duct cyst	M	4
11	Thyroglossal duct cyst	M	3
12	Thyroglossal duct cyst	M	3
13	Pre-auricolar cyst	M	1
14	Pre-auricolar cyst	M	15
15	Gynecomastia	M	10
16	Gynecomastia	M	10
17	Lymphangioma	M	9
18	Lymphangioma	M	7

**Table 3:** Surgical details of mass excision with rubina® Lens.

Case	Intraoperative time (min)	ICG administrated (ml)	Intraoperative complications	Postoperative complications
Nose mass	52	1	No	No
Neck mass	125	5	No	No
Neck mass	134	5	No	Seroma
Thyroglossal duct cyst	47	2	No	No
Thyroglossal duct cyst	64	2	No	No
Thyroglossal duct cyst	70	2	No	Seroma
Thyroglossal duct cyst	58	2	No	No
Preauricular cyst	39	0.5	No	No
Preauricular cyst	43	0.5	No	No
Scalp cyst	35	1	No	No
Scalp cyst	41	1	No	No
Supraorbital cyst	40	0.5	No	No
Supraorbital cyst	43	0.5	No	No
Supraorbital cyst	45	0.5	No	No
Gynecomastia	80	1	No	No
Gynecomastia	80	1	No	No
Lymphangioma	65	1	No	No
Lymphangioma	63	1	No	No

## Discussion

In laparoscopic and robotic surgery, ICG fluorescence imaging is largely used in adult and children to intraoperative decision-making strategy and to guide surgeons, not only during the dissection phase. However, its use in open surgery is still limited. In adults, ICG-mediated fluorescence has been proposed for sentinel lymph node biopsy in breast surgery and for melanoma using a specifically designated camera for “open” surgery (11, 12). In children, open surgery ICG technology assisted until now has seldom been reported (13–18). Surgery for mass removal could be challenging in this age group because sometimes it is difficult to differentiate the mass margins from the surrounding tissues. Singer et al. demonstrated the presence of procedure-related reasons for hospital readmission after pediatric neck mass surgery, including postoperative neck mass, wound issues, and infection (6). As regards gynecomastia, subcutaneous mastectomy through a trans- or peri-areolar access may cause poor scarring and asymmetry with bad cosmetic results (6) and sometimes it is difficult to identify mass margin through a mini sub-areolar incision. For this reason, we decided to adopt Rubina® lens ICG fluorescence technology system for video assisted open mass excision and to report our preliminary experience. Our goal was to provide a measurable or quantifiable method that can objectively assess mass anatomy intraoperatively and reduce the risk of complications and treatment failure due to poor margins visualization, practice variation, and adjacent structures involvement. ICG fluorescence was visualized using a commercial fluorescence imaging system (Rubina® lens; Karl Storz, Tuttlingen, Germany) consisting of a digital video recorder with an integrated near-infrared (NIR) light source (energy 0·16 W, wavelength 780 nm). The object lens of the camera was covered with a filter (835 nm) to collect NIR radiation and reject visible light. During operation, 0.5–1 ml of ICG solution using a 25-gauge needle was performed for every mass, except for gynecomastia cases, where ICG solution was injected intra-dermally into the peri-areolar region of the breast. Based on our experience, we believe that the holding arm system is better when compared to hand-held technique, allowing a more stable vision, and avoiding hand tremor. As for the type of injection, the amounts of product adopted depend on the size of the mass: in general, 0.5–1.0 ml is enough to have a good visualization. As for the way of injection, it is preferable to inject the product through the skin to avoid any leakage of product around that would impair the identification of perilesional margins.

Our preliminary results have showed that the use of ICG for open mass excision was feasible in all the cases. We found this technology of great help in visualizing the anatomy of the masses and their contact with adjacent structures. In all our patients, the plane of dissection was clearly visible and eased mass removal, above all in the case of large neck mass. For thyroglossal duct cysts, we were able to follow the fistulous tract until the cyst thus avoiding the risk of tracheal injury. For gynecomastia, dissection was simplified through the clear visualization of the mass and its demarcation from adjacent adipose and subcutaneous tissue. Intraoperative time did not show any modification for the use of Rubina® lens based on our experience, but a case-control study needs to be run to verify this assumption. In fact, the technology was set before starting the procedure, thus allowing a fast covering of the camera with sterile plastic sheets intra-operatively.

Postoperative complications included seroma formation 3 weeks after surgery in 2 cases (neck mass and thyroglossal duct), which were both managed through aspiration of the fluid in the outpatient setting. These complications are not strictly related to ICG (19).

Surgical pathology reports confirmed complete mass excision in all the cases.

In addition, we found the system easy to set and we believe that it would be useful to adopt it in the operative theater. With a minimum follow-up of 4 months, we reported no other complication and no discoloration of the skin around the site of injection.

In conclusion, based on our experience, Rubina® lens ICG-based fluorescence technology was utilized to provide enhanced visualization of anatomy and margins of the lesion allowing easy mass dissection, low complications rate, time-saving surgery, real time reliability and clinical safety in open surgery procedures. Further cases need to be performed to evaluate the exact role of this technique in open pediatric surgical procedures and more studies involving larger patient cohorts and case-control studies should be advocated to provide stronger results.

## Data Availability

The raw data supporting the conclusions of this article will be made available by the authors, without undue reservation.
